# Innovation Orientation in the Non-profit Sector: Analysis of Its Impact on the Performance of Spanish Non-governmental Development Organizations

**DOI:** 10.3389/fpsyg.2021.797621

**Published:** 2021-12-09

**Authors:** Víctor Valero-Amaro, Clementina Galera-Casquet, María Jesús Barroso-Méndez

**Affiliations:** ^1^School of Industrial Engineering, University of Extremadura, Badajoz, Spain; ^2^Faculty of Economics and Business, University of Extremadura, Badajoz, Spain

**Keywords:** innovation orientation, success, performance, NGDOs, Sustainable Development Goals

## Abstract

Since 2015, the approval of the 2030 Agenda and of the 17 Sustainable Development Goals (SDGs) has led to a notable reshaping and expansion of the architecture of the international cooperation system. The SDGs mark a new path for the planning processes of the different actors working for development, expanding their goals, proposing an update of the roles they must play, and defining new frameworks for relationships and spaces for action. Non-governmental development organizations (NGDOs), whose traditional mission focused on reducing the poverty gap, defending human rights, or promoting environmental protection, must be able to respond satisfactorily to these new challenges, pass beyond their classic positions, and adapt to an increasingly complex and turbulent global context. Achieving high impact in the SDGs requires development organizations to be more agile and innovative. With the intention of bringing visibility to the importance that innovation can play in the success and results achieved by development cooperation organizations, the main objective of this study was to validate, through a sample of Spanish NGDOs, a causal model that represents the positive contribution which innovation orientation generates in the result of the activity of these entities. This research concludes by confirming that innovation orientation favors the attainment of a higher degree of success in the projects and actions carried out by non-profit entities which promote the SDGs, which has a direct and positive impact on the performance they achieve.

## Introduction

In the current economic and social context, the survival of organizations is increasingly complex. The environment has dramatically transformed, forcing organizations to have greater management skills, essential among which is the capacity to innovate (Areed et al., 2021; Brand et al., 2021). Organizational success requires greater competitiveness for organizations in the form of innovation (García-Zamora et al., 2013; Xie et al., 2021).

Innovation refers to the ability to create new product, or service, or develop a new organizational structure or administrative system (Damanpour, 1991). It refers to carrying out new processes and providing new products to provide stakeholders with a distinguished value (Obeidat et al. 2021).

According to Bessant et al. (2005), innovation represents the fundamental renewal process in any organization. In fact, this process is absolutely extensible to and recommendable for non-governmental organizations specialized in the field of development (NGDOs; Díaz-Perdomo et al., 2021). Although the international cooperation for development sector has always been characterized as a dynamic sector under constant review, the arrival of the 2030 Agenda and the beginning of the period of validity of the Sustainable Development Goals (SDGs) have meant an important change in the paradigm of development cooperation. The traditional approach to cooperation, focused especially on the fight against poverty, has expanded with new fundamental aspects, such as the reduction of inequalities, the promotion of sustainable and responsible production and consumption models, the fight to promote joint action for the climate, the conservation of biodiversity, the creation of healthy living spaces, and creating peace. The new architecture of the SDGs has posed significant challenges to all stakeholders, both public and private, and both the aid channeling and the aid recipient state and offer a new understanding of the global development partnerships that are necessary to achieve this (Elson and Balakrishnan, 2013). NGDOs are not alien to all this, and such aspects as the role they play, the goals they pursue, the geographical spaces in which they work, or the relationship frameworks they have are subject to debate and redefinition (Coordinadora Española de ONGD - CONGDE, 2015). The ambitious challenges of the SDGs require agile and innovative organizations to the extent that innovation, specifically social innovation, is now seen as a solution to the growing global problems (Anheier et al., 2019; Díaz-Perdomo et al., 2021).

The concept of social innovation has been conceptualized by numerous authors and from different perspectives. However, most of the literature agrees that a social innovation process contains four characteristics (Howaldt and Schwarz, 2010; Grimm et al., 2013; Anheier et al., 2014; Van der Have and Rubalcaba, 2016; Díaz-Perdomo et al., 2021): its objectives – attempting to address important social or environmental challenges, its means – as it involves a collaborative process which involves all stakeholders, its long-term orientation – keeping a focus on the sustainable use of resources, and its final consequences and impact, as it pursues changes in social practices until a systemic change is achieved.

The ability of NGDOs to drive social innovation, and thus contribute to the achievement of the SDGs, is inherent to their own mission and derives from the role they play in connecting their beneficiaries with the civil society they represent. Indeed, NGDOs can foster all four dimensions that make up a social innovation. First, since their essence is to address social needs, their mission incorporates the social dimension specified in the SDGs. Second, as entities representing civil society, they often maintain close communication channels with the beneficiaries of their programs. Third, NGDOs, unlike companies, tend to maintain a long-term results orientation, as they are oblivious to the competitive pressures experienced by the business sector. And fourth, non-profit organizations (NPOs) tend to pursue systemic change as the ultimate goal of their projects.

Based on the reality of the NGDO sector and the proposed definitions of innovation, the general objective of the present study was to analyze the important relationship that can exist between innovation and good performance for development entities. Specifically, in order to achieve this goal, the following specific objectives were established as: to design a causal model that links innovation orientation with success and performance; to design – or to adapt to NGDO context – and to validate the measurement scales to obtain information on the variables of the model; and finally, through a sample of Spanish NGDOs, to test whether innovation orientation contributes positively to the results these entities achieve in the development of their activity.

Accordingly, the structure of the study is as follows. In the first place, the concept of innovation orientation is addressed in detail, setting forth the different conceptualizations proposed so far, and analyzing the antecedents and results of this construct that have been recognized by different authors who have studied this factor. Second, a specific scale of innovation orientation adapted to the field of non-profit organizations is proposed, based on previous research. Next, a structural model is proposed in which the relationship that innovation orientation has with the success of NPOs and in turn the impact that these two elements generate on the performance of these entities are analyzed. Finally, from the data obtained from the sample of NGDOs, the measurement instruments are validated, the proposed structural model is assessed, and the model’s predictive capacity to foretell the performance of NGDOs from the level of innovation orientation they achieve is analyzed.

## Conceptualization of the Innovation Orientation Construct

In 2009, and in order to provide a simple textual definition that could act as a basis to summarize the essence of innovation, Baregheh et al. (2009) carried out a systematic review of the then existing literature about this concept. After analyzing more than 60 definitions of innovation from different disciplines, they proposed that innovation is a multi-stage process by which organizations transform ideas into new or improved products, services, or processes in order to successfully advance, compete, and differentiate themselves in their markets (Baregheh et al., 2009).

This definition especially highlights the importance of innovation, which must necessarily be carried out through product innovation, for organizations to achieve greater results. Therefore, it is not surprising that currently, the degree of innovation orientation that organizations are able to maintain, especially those organizations that carry out their activity in dynamic environments, has become a matter of great importance and a key factor to achieving success.

The term innovation orientation has frequently been used in the innovation literature, although with a diversity of conceptualizations and meanings (Siguaw et al., 2006). Following a chronological order of the most used definitions that also have greater repercussion in the literature, initial mentioned should be made of Manu (1992) for whom innovation orientation covers all the innovation programs of companies, by being an activity strategic in nature as it provides direction to deal with the markets.

Manu and Sriram (1996) conceptualize the term innovation orientation as a multiple construct made up of three components: introduction of new products, R&D expenditures (products and processes), and order of entry into the market. For these authors, a combination of these elements offers a better reality of an organization’s capacity for innovation than do single-element approaches. On the one hand, the component of introduction of new products reflects the outputs that result from R&D efforts or expenditures. They consider the inclusion of this factor to be necessary because, although patent registrations also reflect innovative outputs, a significant number of them are not actually commercialized. Therefore, this component turns out to be a more effective measure of output. On the other hand, the component of market entry order analyzes the position of a company in the market at the moment a given product is launched on the market. Thus, to the extent that an organization is one of the first to develop a particular product or service, it may be classified as being more innovative than another.

Hurley and Hult (1998), however, conceptualize innovation orientation as the openness to new ideas as an aspect of the culture of a company. Accordingly, the innovation contribution that can come from the organizational culture itself is the key measure of the organization’s innovation orientation, and it is not necessary to add more measurement elements.

In contrast, Worren et al. (2002), in line with previous works, defined innovation orientation as a construct made up of two components: strategic effort (which supposes a link between the possibilities of product improvement and the strategic intention of the companies to develop new products or to enter new markets) and a climate of innovation, which implies the creation of an environment where the development of new ideas is encouraged and rewarded, and where employees maintain a shared mission.

For Siguaw et al. (2006), innovation orientation can be understood as a multidimensional structure of knowledge composed of a learning philosophy, strategic direction, and “transfunctional” beliefs. These, in turn, guide and direct all the strategies and actions of the organization, including those embedded in the formal and informal systems, behavior, competencies, and processes of the company to promote innovative thinking and facilitate the successful development, evolution, and execution of innovations. As can be seen in this definition, for these authors innovation orientation is made up of three components (Siguaw et al., 2006):

1. A philosophy of learning. One of the requirements for a company to be innovation-oriented is a philosophy of learning, defined as a generalized set of understandings across the organization about learning, thinking, acquiring, transferring, and using knowledge in the company to innovate (Siguaw et al., 2006). This component of innovation orientation should enable various functional units within a company to learn and apply knowledge in a self-reinforcing cycle from various sources, including past experience.
2. A strategic direction. This component of innovation orientation can be understood as the strategic beliefs and understandings that define who the company is and how the organization’s activities are assembled to ensure that innovation occurs at the right moment (Siguaw et al., 2006). Therefore, strategic direction implies clarity of thought and purpose, in general articulated through statements and objectives of vision and mission.
3. A complementary transfunctional climate. A final requirement for a company to be innovation-oriented is a cross-functional acclimatization, defined as an integrated knowledge structure that encourages and facilitates the transfer of knowledge between and within the subunits, to preserve the diversity of points of view, and foster cooperative beliefs and understandings among all the functional areas to direct them toward innovation (Siguaw et al., 2006). Therefore, transfunctional acclimatization is the process that allows the organization to capture and complement departmental thoughts, plan how functional knowledge structures should be used to learn and develop strategies as a global company, understanding that this facilitates innovation within the organization.

Talke et al. (2011) conceptualize innovation orientation through two types of orientation to strategic innovation which captures the core of innovation. One is proactive market orientation, which represents the needs of emerging and non-articulated clients (Narver et al., 2004; Talke et al., 2011). The other is the orientation to proactive technology, which symbolizes a search for opportunities that lead companies to act in anticipation of future demand by experimenting with change, the exploitation of emerging opportunities, and the application of the latest technologies in the development of new products.

Finally, it is important to highlight the recent definition by Norris and Ciesielska (2019) who conceptualize innovation orientation as “a multiple construct with a focus on driving innovation-based practices and values throughout the organization primarily through four core aspects: culture, flexibility in structures, capital and knowledge capabilities and understanding environmental dynamics with the aim of driving positive organizational performance” (Norris and Ciesielska, 2019, p. 126).

As can be seen, the definitions of the construct innovation orientation (Table 1) address different aspects and reveal a lack of general consensus about its different components or elements, since there is no single or predominant point of view about its real meaning. For this study, it has been considered appropriate to follow the definition proposed by Hurley and Hult (1998) which is focused on the cultural predisposition of organizations to innovate, over other views more oriented to emphasizing the innovative behavior of companies. It is also a conceptualization that has been used with very good results in recent research (Chen et al., 2009; Zhang and Duan, 2010; Theodosiou et al., 2012; Zhang et al., 2015).

**Table 1:** Definitions of innovation orientation.

Manu (1992)	Innovation orientation, being strategic in nature, encompasses all of a firm’s total innovation programs since it provides direction for dealing with the markets.
Manu and Sriram (1996)	Innovation orientation is a three-component construct: introduction of new products, expenditure on RandD (products and processes), and order of entry into the market.
Hurley and Hult (1998)	Innovation orientation implies that one of the aspects of the firm’s culture is openness to new ideas.
Worren et al. (2002)	Innovation orientation is a two-component construct: business strategic intent and climate of innovation.
Siguaw et al. (2006)	Innovation orientation is a multidimensional knowledge structure. It comprises a learning philosophy, a strategic direction, and cross-functional beliefs which, in turn, guide and direct all of the firm’s strategies and actions, including those embedded in the firm’s formal and informal systems, behaviors, skills, and processes. Together they foster innovative thinking and facilitate the successful development, evolution, and execution of innovations.
Talke et al. (2011)	Innovation orientation is a construct made up of two types of strategic innovation orientation: proactive market orientation, and proactive technology orientation.
Norris and Ciesielska (2019)	Innovation orientation is a multiple construct with a focus on driving innovation-based practices and values throughout the organization primarily through four core aspects: culture, structure flexibility, capital and knowledge capabilities, and understanding environmental dynamics with the aim of driving positive organizational performance.

## Background and Consequences of Innovation Orientation

The existing literature, mainly in the business field, has mentioned that an organization’s innovation orientation depends on its having of different key attributes. Although the number of studies focused on the study of these attributes is still limited, the following are those antecedents for which there is greater evidence of their influence (Table 2).

**Table 2:** Main antecedents of innovation orientation.

Market orientation	Han et al. (1998) Kirca et al. (2005) Tajeddini et al. (2006) Grinstein (2008) Camarero and Garrido (2008) Garrido and Camarero (2010) Dibrell et al. (2011) Rahab (2012) Algarni and Talib (2014) Choi (2014)
Relationship orientation	Human and Naudé (2010) Zehir et al. (2011)
Learning orientation	Mullen and Lyles (1993) Nasution et al. (2011) Rahab (2012)

As can be seen in Table 2, numerous authors, in the business context (Han et al., 1998; Kirca et al., 2005; Tajeddini et al., 2006; Grinstein, 2008; Dibrell et al., 2011; Rahab, 2012) and the non-profit context (Camarero and Garrido, 2008; Choi, 2014), have postulated that an organization’s innovation orientation depends on its degree of market orientation. Thus, for example, in the business environment, Dibrell et al. (2011) argued that market-oriented companies have a greater propensity to satisfy current and future needs of their customers. In this way, a market-oriented company is in a position to more effectively develop better incremental or radical innovations. In other words, market-oriented companies can use their acquired knowledge to change products and processes in order to meet the changing and latent needs of their customers, as well as identify new potential customers.

This approach has also been studied, although with much less frequency, in the non-profit context (McDonald and Srinivasan, 2004; Weng et al., 2011). The results of the studies by Camarero and Garrido (2008) and Garrido and Camarero (2010) confirm that a market orientation is very important for the development of technological and organizational innovations in museums. Modi and Mishra (2010) demonstrated the relationship between the market orientation of non-profit entities in India and their innovation orientation. More recently, Choi (2014) gave a partial contrast of the relationship between market orientation and innovation with a study about community centers in Korea.

Although their presence in the literature is notably less than that detected in the case of market orientation hand, other antecedents have also been analyzed as causes of innovation orientation. Thus, several workers have shown that the degree of learning orientation of an organization positively influences its level of innovation orientation. They suggest that a sustained orientation toward organizational learning will improve the efficiency and efficacy of a company’s innovative activities (Mullen and Lyles, 1993). Finally, it should be mentioned that some authors have highlighted as an antecedent of innovation orientation the organization’s level of orientation to relationship. Thus, Human and Naudé (2010) have proposed and corroborated empirically that relationship orientation, measured by four sub-constructs (trust, social bonding, shared values, and reciprocity), exerts a positive impact on the innovation orientation of the organization.

Once the most important antecedents of the innovation orientation have been presented, the results of this construct that have been most referenced in the literature reviewed will be analyzed in detail, mainly due to its greater development in the business context (Table 3). As can be seen in the table, most of the studies reflect the importance of innovation orientation for the organization to achieve greater performance (Manu, 1992; Han et al., 1998; Human and Naudé, 2010; Algarni and Talib, 2014; Datta et al., 2019; among others). Thus, Han et al. (1998) argued that the link between innovation orientation and performance is attributable to the fact that innovations serve to adapt to the uncertainties (technological or market turbulence) that a company has to face in its entrepreneurial environment. For their part, Zhou et al. (2005) showed that technical and market innovations positively influence performance, although the former have the more profound impact. Finally, Theodosiou et al. (2012) pointed out that companies with a greater innovation orientation respond more effectively to their environment and develop new marketing capabilities that lead to obtaining greater competitive advantages and improved performance. Accordingly, the expectation was to find that in the context of NGDOs this relationship could also be contrasted, and the following hypothesis was proposed as:

**Table 3:** Principal results of innovation orientation.

Performance	Manu (1992) Deshpandé et al. (1993) Manu and Sriram (1996) Han et al. (1998) McDonald and Srinivasan (2004) Zhou et al. (2005) Zhou et al. (2009) Camarero and Garrido (2008) Garrido and Camarero (2010) Human and Naudé (2010) Modi and Mishra (2010) Zehir et al. (2011) Weng et al. (2011) Algarni and Talib (2014) Choi (2014) Ionescu and Ionescu (2015) Ganesan and Sridhar (2016) Farooq (2017) Wang et al. (2019) Datta et al. (2019) Mohedano-Suanes et al. (2021) Farooq et al. (2021)
New product success	Narver et al. (2004) Zhang and Duan (2010) Chou and Yang (2011) Zhang et al. (2015) Gundry et al. (2016) Stock and Schnarr (2016)
Marketing capabilities	Theodosiou et al. (2012)

*H1*: The innovation orientation in NGDOs positively and directly affects the performance that these organizations achieve.

Various authors (for example, Zhang et al., 2015) have argued that innovation orientation is the key driver to overcoming obstacles and improving a company’s ability to successfully adopt or implement new systems, processes, or products, due to, among other aspects, the fact that companies with a greater innovation orientation dedicate more resources to R&D activities, which encourages the successful development of products and services. In another sense, it is interesting to note that innovation orientation, in addition to being proposed as a mediating construct between different antecedents and results, has also been identified as a moderating construct (Zhang and Duan, 2010; Gundry et al., 2016). Thus, for example, Zhang and Duan (2010) proposed that innovation orientation positively moderates the market orientation/new product success link, arguing that companies with a greater innovation orientation are more likely to use the information and knowledge they have about their customers and competitors in a more creative way, thus producing products and services with a greater likelihood of success in their different target markets. This evidence of the relationship between innovation orientation and new product success suggests the following hypothesis:

*H2*: An innovation orientation influences the success of new projects developed by NGDOs, having a positive and direct influence.

With respect to the success of new products or projects, success has been a diffuse concept in the literature, and there is a lack of consensus as to its definition. On the one hand, in the context of NPOs, the success of their activity has been equated in some cases with value creation (Austin, 2010; Murphy and Arenas, 2010; Flores et al., 2011; Austin and Seitanidi, 2012a,b), or with the achievement of higher performance (Graf and Rothlauf, 2012; Stadtler, 2012), or the achievement of social change or transformation (Seitanidi, 2010; Sakarya et al., 2012) inherent to their mission. In business, one can find two-dimensional approaches to measuring success, conceptualizing such term mainly through two dimensions, goal fulfillment and satisfaction (Arenas, 2003; Kauser and Shaw, 2004; Arenas and García, 2006), and one-dimensional approaches that equate success with the achievement of higher performance (Baker and Sinkula, 1999, 2009; Song et al., 2000; Sarkar et al., 2001; Hunt et al., 2002; Ariño, 2003; Tser-Yieth, et al., 2009). Within this last group of works, performance has also been measured in different ways. Thus, for example, authors, such as Ariño (2003) have used six performance measures: fulfillment of objectives, satisfaction, indirect effects, longevity, contractual changes, and survival. Other authors have used four measures related to goal attainment, spillovers, relative profitability, and overall performance (Parkhe, 1993). And finally, some researchers have considered it appropriate to use only three dimensions: market share, overall performance, and new product success (Baker and Sinkula, 1999).

This paper addresses the line followed by previous studies (Song and Parry, 1997; Atuahene-Gima and Evangelista, 2000; Narver et al., 2004; Atuahene-Gima et al., 2005; Baker and Sinkula, 2005; Calantone et al., 2006; Zhang and Duan, 2010; Kam and Tong, 2012; Kock, 2015; Zhang et al., 2015), in which new product success is not analyzed as a measure of firm performance, but as a completely independent construct, although directly related to the latter. Under this approach, the literature shows that the success of new products is positively related to the performance of the organization (Montoya-Weiss and Calantone, 1994; Langerak et al., 2004; Baker and Sinkula, 2005; among others). The reason behind this relationship is that, today, organizations face higher levels of competition, changing environments, higher rates of technical obsolescence, and shorter product life cycles. Under these conditions, the development of successful products considerably reduces the uncertainty existing in business environments, which leads to greater organizational performances (Langerak et al., 2004). Accepting this reasoning, it was proposed to extend this logic to the field of NPOs, and a new research hypothesis was formulated that relates success in new projects or actions with the general performance of non-profit entities.

*H3*: Success in the development of new projects or actions favors and positively contributes to the performance achieved by NGDOs.

**Figure 1:**
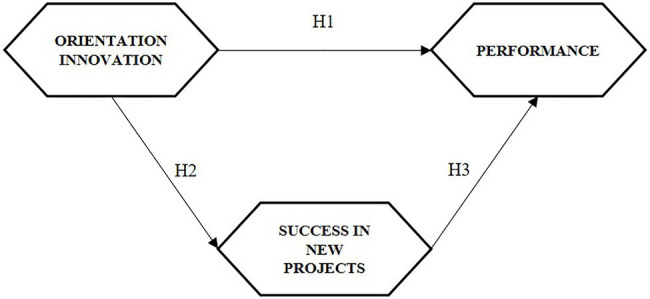
Proposed research model.

From the proposed research hypotheses, the model shown in Figure 1 was constructed.

Finally, it is worth mentioning that previous studies have suggested that the relationship between innovation orientation and performance may not be direct but rather mediated by other variables (Hult et al., 2004). The present study proposes to test whether there is a significant mediating effect in the relationship between innovation orientation and the performance of NGDOs, an effect caused by the mediation generated by the success of new projects.

*H4*: The relationship between innovation orientation and performance is mediated by the success in the development of new projects or actions.

## Materials and Methods

### Data Collection and Sample Selection

In the design of the empirical research, the NGDO legally constituted within the Spanish territory were chosen as the units of analysis. Specifically, the target population was defined as those NGDOs that are associated or federated with some NGDO Coordinator (either state or regional). A questionnaire was sent to all the entities that were part of the said target population, so no specific sampling strategy was carried out with the aim of selecting sample units within the population. The final sample was thus made up of those NGDOs that responded to the request for collaboration in this research. Therefore, it is assumed that the sampling procedure carried out was a non-probabilistic convenience sampling, this being a common method in this type of study where, due to its small size, the entire target population can be accessed. The lower limit was established to obtain at least 80 entities surveyed. This value guaranteed the appropriateness of the statistical techniques that were planned to be carried out.

Thus, starting from a target population of 842 registrations – total number of NGDO associated with the Spanish Development NGO Coordinator, or with one of the 17 Regional Development NGO Coordinator–- a first wave of invitations *via* e-mail was sent to all these organizations. Of these e-mails sent, approximately 150 were returned, which led to the final sample of around 692 organizations. Several days after the launch of the questionnaire, in order to improve the response rate and be able to reach the minimum sample set, a reminder was made *via* telephone with those NGDOs affiliated with the Spanish NGDO Coordinator who had not yet answered and who had not expressed their refusal to collaborate in the study. In the case of NGDOs associated with Regional Coordinators, a new e-mail was sent, recalling the invitation to participate in the study. The deadline for receiving questionnaires was closed, having obtained a total of 104 duly completed questionnaires, which represented a response rate of 15.02% of the real population of identified NGDOs. During the opening period of the electronic questionnaire, a total of 496 visits to the questionnaire were registered, of which 91.3% accessed through a direct link, 7.7% through Facebook, and 1% through an invitation by mail. Of this total number of visits, the questionnaire was completed 20.8% of the times, while 72.2% of the visits only viewed the questionnaire, and 6.9% left it incomplete.

An analysis was carried out to find out if there were any bias in the responses due to having made a recall among the population, and therefore having captured the data in two different waves. For this, the procedure recommended by Armstrong and Overton (1977) was followed. Thus, the responses obtained after the first and second requests were compared by means of a test of differences of means on all the variables included in the study, suggesting the multivariate analysis of variance (MANOVA) as a method to assess non-response bias. The results of the MANOVA did not show any significant differences between the subsamples, and it can be deduced that there were no significant differences in the responses obtained regardless of the wave in which they were collected.

Finally, in order to guarantee the internal validity of the research, the questionnaires were sent recommending them to be responded to by a person with high responsibilities in the management of the organization, such as people who are directors, the secretariat general, and manager (Modi, 2012), so that their knowledge of the organization is high, and not only limited to a certain department, regional area, or program (Kohli and Jaworski, 1990), as the information available to senior positions of responsibility about policies, actions, organizational orientation, or results is more complete (Norburn and Birley, 1988). Approximately 89% of the responses came from people with a representative position in the NGDO or headquarters in which they worked (direction, management, presidency, vice-presidency, member of the board of directors, etc.), and only 11% of the responses were completed by people who held more specific responsibilities in a specific area or department of the NGDO. The rest of the characterization of the sample is shown in Table 4.

**Table 4:** Characterization of the sample.

Variables	N° of organizations
N	104
Size (by project expenditure)	
*Small*	58%
*Medium*	28%
*Large*	13%
*Very large*	1%
Age of the organization (years since its founding)	
*Less than 10 years*	3%
*Between 10 and 20 years*	33%
*Between 20 and 30 years*	48%
*Over 30 years*	16%
Geographical scope (presence of open offices)	
*Local*	38%
*National*	41%
*International*	21%

### Measurement Scales

To measure the innovation orientation, the main scales used in previous studies were analyzed, finding the study by Hurley and Hult (1998) to especially stand out. In this sense, studies that address the innovation orientation in the non-profit sphere (Modi, 2012) have also been based on this scale for the construction of a measurement instrument that reflects this reality. In its final version, this scale presents five items, although from its reading it can be seen that some of them may be difficult to apply in the field of development organizations. Therefore, in order to complete the scale, we also considered the studies of Chen et al. (2009), whose research presented an innovation orientation measurement scale that initially also started from Hurley and Hult (1998), and Gundry et al. (2016), who developed an innovation orientation measurement instrument based on the preliminary research done by Koys and DeCotiis (1991). From these sources, an original innovation orientation scale was designed that contained 14 indicators.

The construct of success in new project development was measured on the basis of the five-item scale proposed by Baker and Sinkula (1999), but with changes to the wording of its indicators to adapt it to the context of NGDOs. Together with this, an item was included from the scale developed by Kam and Tong (2012) in turn adapted from that used by Narver et al. (2004) to reflect the perception of success rate in relation to other similar organizations. In synthesis, the original scale of success in the development of new projects consisted of six items.

With respect to the performance of NGDOs, previous performance scales with strong links to financial results have been avoided, due to their limited applicability, as the only performance indicators in non-profit organizations (Wood et al., 2000; Camarero and Garrido, 2008; Mahmoud and Yusif, 2012; Pinho et al., 2014). Lamb and Crompton (1990) considered it appropriate to assess the results of NPOs through two fundamental measures: efficacy and efficiency. Other authors have taken a focused view of performance, using only indicators related to fund-raising (Bennett, 1998; Brady et al., 2011). Balabanis et al. (1997), on the other hand, considered it appropriate to measure performance through four key measures: the achievement of short-term objectives, the achievement of long-term objectives, the number of volunteers, and the ratio between the contribution of donors and the expenses of the NPO. In other studies, such as those carried out by Gainer and Padanyi (2002) or Padanyi and Gainer (2004), performance has been operationalized using three fundamental dimensions: growth in customer satisfaction, growth in the acquisition of resources, and growth in the level of reputation among organizations in the sector. Modi and Mishra (2010) measured performance using four dimensions: the three proposed by Gainer and Padanyi (2002), that is, customer satisfaction, acquisition of resources, and level of reputation, and one of the dimensions contemplated in the study by Lamb and Crompton (1990), specifically efficacy as a measure of the ability of the NPO to achieve its organizational mission.

Sanzo et al. (2015), in a study that analyzed the success of the association processes between companies and NPOs, went in depth into this vision of performance by developing a performance scale in NPOs that brought together some of the different views expounded on above. Specifically, their approach to the concept of performance suggested that this reality is made up of four key dimensions: the degree of fulfillment of the NPO’s mission, the achievement of certain operational results, the fulfillment of results in raising funds, and the visibility of the NPO. The construction of the performance scale for NGDOs has followed this proposal and considers four dimensions of performance: impact (measure of the degree of fulfillment of the organization’s mission), operational efficiency (measure of the ability to implement the projects and actions that it designed), private fund-raising (a measure of the effectiveness of raising funds not directly related to projects, commonly called non-finalist funds), and visibility (a measure of the entity’s presence and public exposure). Initially, this scale was created with 10 items that contained the information of these four dimensions.

With the initial scales, a process of validation and improvement of these measurement tools was proposed through consultation with a panel of experts (professionals from development cooperation organizations, academics, and technicians from NGO coordinators) made up of 12 people. Two rounds of consultations were carried out in which the validation of each dimension or indicator was requested, as well as proposals for improvement, rejection, or inclusion of new indicators. As a final result of the expert consultation process, the innovation orientation scale was finally made up of eight indicators, since six of those initially proposed were rejected as it was considered that they did not adjust to the reality of development organizations. In the case of the scale of success in new projects, the original wording of the indicators was modified and one of them rejected, thus ending up with five items. With respect to the performance scale, the 4D structure was maintained and new indicators were incorporated based on the contributions of the panel of experts, resulting in a scale made up of 15 indicators.

With respect to the nature of the constructs, both the innovation orientation and the success in new projects were modeled as first-order composite models (Bollen and Bauldry, 2011; Bollen., 2011; Henseler, 2017), i.e., one-dimensional composite models that responded to a construction in which the relationship between the construct and the indicators did not necessarily respond to a cause-effect relationship but, on the contrary, the indicators were components of the construct that represent different facets of it (Sarstedt et al., 2016). Although the modeling of composites did not require any assumption regarding the correlation between indicators of the same dimension, it was assumed that there may be a correlation between the indicators of the same construct, which allowed the scales to be modeled as Mode A composite models (Henseler et al., 2014; Sarstedt et al., 2016). Performance was modeled as a Mode A second-order composite model composed of four dimensions (impact, operational efficiency, fund-raising, and visibility), and assuming the existence of a correlation between these dimensions. In turn, each of these performance dimensions were also modeled as Mode A composites.

## Results

The assessment of the measurement models and the structural model was developed by Structural Equation Modeling (SEM). This type of modeling can be executed through two different approaches – methods based on the analysis of covariances for which software, such as LISREL, EQS, or AMOS, can be used, or methods based on an analysis of variance or partial least squares (PLS) for which there are statistical program packages, such as PLS-Graph or Smart PLS. Specifically, the present research used the last of these methods, the PLS technique, through the Smart PLS v3.3.3 program (Ringle et al., 2015). The main reason that led to the use of PLS was the modeling of constructs as composites (Sarstedt et al., 2016; Rigdon et al., 2017). Along with this, the exploratory nature of the research (as there is not enough previous literature about the relationships analyzed in the study’s context), the interest in knowing the predictive capacity of the model, and the advantage of obtaining aggregate scores of the compounds (to model from them the second-order construct) justified the use of PLS as an analysis tool (Henseler et al., 2016; Henseler, 2018).

As a preliminary step to the analysis of the measurement instruments of the model, a study of missing data and outliers was carried out. The criterion adopted for the identification of cases with high missing value levels was those that presented more than 10% of missing data. In those cases which did not reach this value, the missing values were replaced by the mean of the variable (Hair et al., 2007). With respect to outliers, a multivariate detection was performed on each scale using the Mahalanobis distance through linear regressions, taking as a criterion the elimination of those indicators whose probability of presenting an associated distance for random reasons was less than 0.001. As a result, three cases were eliminated, establishing the definitive sample of 101 study elements.

### Common Method Bias

In an SEM model, common method bias (CMB) is a phenomenon caused by an incorrect design of the measurement method (McGonagle, 2017). CMB can lead to artificial variations in the relationships between variables (Cernas et al., 2017; Kock, 2017; Malhotra et al., 2017), as the data collected do not accurately reflect the actual opinion of the sample surveyed. To avoid this bias, the questionnaire was drafted following the indications of Podsakoff et al., 2012. Additionally, a collinearity test based on variance inflation factors (VIF) was performed to detect the presence of CMB (Kock, 2015). A VIF above 3.3 would indicate the existence of collinearity, and thus, that the model may be affected by CMB (Kock, 2015; Rubio et al., 2019). The model does not include any VIF greater than 2.4 and can be considered free of CBM.

### Assessment of the First-Order Model

A first assessment step is related to the properties of the first-order measurement instruments, taking into account that one of the constructs (performance of the NGDO) is modeled as a second-order construct. This assessment was carried out in terms of individual reliability of the items, construct reliability, and convergent validity of the scales. With respect to individual reliability, the decision was made to maintain those indicators that reached a loading (communality) greater than 0.6. This criterion is justified because they are scales generated specifically for this context (Barclay et al., 1995) and so as to try not to compromise the validity of the content (Hair et al., 2011). Nonetheless, the indicators with very low loadings were eliminated, with one indicator being eliminated from the innovation orientation scale and another from the performance indicators. In the final result, only five indicators presented a loading greater than 0.6 but less than 0.707 (Carmines and Zeller, 1979). With respect to construct reliability (internal consistency of each scale) and convergent validity, all scales reached optimal values. The results of the analysis of the measurement scales of the first-order model are presented in Table 5.

**Table 5:** Full collinearity VIF. CMB analysis.

Variables	Innovation Orientation	Success	Performance
VIF	1.460	2.400	2.147

Once the validity of the first-order scales had been verified, the aggregate scores of the performance dimensions were used for the construction of the second-order model, where performance is in turn composed of four dimensions.

### Assessment of the Second-Order Model

The final second-order model was assessed for its measurement instruments in terms of the individual reliability of the indicators, construct reliability, convergent validity, and discriminant validity. Table 6 presents the results of the assessment of the second-order model measurement instruments in terms of individual reliability, construct reliability (where a CR > 0.7 is required), and convergent validity (which requires an AVE > 0.5). Satisfactory results were achieved in all these requirements. Table 7 presents the results of the discriminant validity analysis. It can be affirmed that the proposed model contains measurement scales that exceed the required psychometric properties, and therefore, it was appropriate to continue with the assessment stage of the structural model.

**Table 6:** Validation indicators of the first-order model’s measurement instruments.

**CONSTRUCT**/Dimension/INDICATOR		Mean	SD	Loading (*λ*)	CR	CR Int_2.5%_	CR Int_97.5%_	Rho_A	AVE
**PERFORMANCE**									
*Impact*					0.864	0.749	0.920	0.794	0.613
PERFORM_1	Degree of fulfillment of my NGDO’s mission and objectives	4.09	0.631	0.811					
PERFORM_2	Impact of executed projects	4.03	0.751	0.788					
PERFORM_3	Satisfaction of my collaborators (partners and donors) in their expectations of my NGDO’s activity	4.01	0.790	0.787					
PERFORM_14	Satisfaction with the activity carried out by my workers and volunteers	3.97	0.764	0.746					
*Private fund-raising*					0.890	0.823	0.926	0.761	0.802
PERFORM_5	Number of my NGDO’s partners and collaborators (private donors)	2.76	1.026	0.883					
PERFORM_8	Volume of private funding (fees, donations, sponsorships, etc.) obtained	2.64	1.122	0.908					
*Operational efficiency*					0.885	0.828	0.918	0.843	0.659
PERFORM_7	Volume of public funding obtained	3.13	1.087	0.736					
PERFORM_9	Volume of total income reached by my NGDO	3.20	0.901	0.804					
PERFORM_10	Number of new projects approved or actions implemented	3.37	1.087	0.906					
PERFORM_11	Number of beneficiaries of our projects	3.79	0.871	0.791					
*Visibility*					0.824	0.759	0.862	0.721	0.540
PERFORM_6	Number of volunteers who collaborate with my NGDO	3.16	1.051	0.683					
PERFORM_12	Number of our website’s visitors and/or social network followers	3.33	1.082	0.799					
PERFORM_13	Presence of my NGO in the media	2.75	1.138	0.727					
PERFORM_15	Degree of active participation in networks	3.50	1.096	0.725					
**SUCCESS IN NEW PROJECTS**					0.864	0.775	0.913	0.845	0.564
SUCCESS_1	Compared with other similar NGDOs, we think our success rate in developing new projects, actions, or campaigns is satisfactory	5.65	1.009	0.801					
SUCCESS_2	We are satisfied with the number of new projects and new actions or campaigns that we identify	5.36	1.223	0.875					
SUCCESS_3	We are satisfied with the success rate of our actions, in relation to our largest competitor	5.43	1.213	0.608					
SUCCESS_4	The public perceives us as an NGDO different from others	4.62	1.508	0.672					
SUCCESS_5	We think that our projects or campaigns serve as a referent for other NGDOs when they design their actions	4.97	1.222	0.769					
**ORIENTATION TO INNOVATION**					0.923	0.889	0.945	0.908	0.635
INNOV_OR_1	Our NGDO pays a lot of attention to innovation	4.95	1.424	0.746					
INNOV_OR_2	We believe in the need to develop new processes and use new resources in the fight against poverty and in education for development	5.77	1.176	0.670					
INNOV_OR_3	We encourage our staff to freely raise and share new ideas, even if they do not work out in the end.	5.85	1.238	0.818					
INNOV_OR_4	In our NGDO, we encourage the search for new solutions to the problems that arise	5.85	1.147	0.908					
INNOV_OR_5	In our NGDO, we often discuss new ways of doing things	5.64	1.354	0.853					
INNOV_OR_6	Our management team actively seeks innovative ideas applicable to our NGDO or our projects	5.50	1.347	0.860					
INNOV_OR_8	In our NGDO, innovation is easily incorporated into project identification and management	4.77	1.296	0.683					

**Table 7:** Validation indicators of the second-order model’s measurement instruments.

**CONSTRUCT**/Dimension/INDICATOR	Loading (*λ*)	CR	CR Int_2.5%_	CR Int_97.5%_	Rho_A	AVE
**PERFORMANCE**		0.857	0.807	0.892	0.813	0.601
*Impact*	0.827					
*Private fund-raising*	0.658					
*Operational efficiency*	0.815					
*Visibility*	0.789					
**SUCCESS IN NEW PROJECTS**		0.865	0.786	0.904	0.842	0.565
SUCCESS_1	0.794					
SUCCESS_2	0.873					
SUCCESS_3	0.616					
SUCCESS_4	0.684					
SUCCESS_5	0.765					
**INNOVATION ORIENTATION**		0.923	0.893	0.942	0.908	0.635
INNOV_OR_1	0.754					
INNOV_OR_2	0.670					
INNOV_OR_3	0.811					
INNOV_OR_4	0.905					
INNOV_OR_5	0.857					
INNOV_OR_6	0.864					
INNOV_OR_8	0.683					

### Assessment of the Structural Model

According to Henseler et al. (2016) and Benítez et al. (2020), the starting point of the assessment of a model should be the analysis of its goodness-of-fit. If the model does not fit acceptably, the data contain more information than the model provides, and therefore, it is not valid to draw conclusions, or these are questionable. Nonetheless, there is some discussion about the appropriateness of the goodness-of-fit analysis. Henseler (2018) himself pointed out that the analysis of goodness-of-fit only makes sense in confirmatory purpose analysis and is inappropriate in studies with predictive purposes or models that include compounds, since the goodness-of-fit is measurable specifically in common factor models (classic reflective constructs).

In the present study, the analysis of the structural model assessed the non-presence of collinearity in the model, the explanatory capacity of the dependent variables, the meaning and significance of the path coefficients, the effect size, and the goodness-of-fit. All these results are presented in Table 8.

**Table 8:** Discriminant validity analysis – second-order model.

*Fornell-Larcker criterion*			
	Innovation Orientation	Performance	Success
INNOVATION ORIENTATION	**0.797**		
PERFORMANCE	0.472	**0.775**	
SUCCESS	0.552	0.726	**0.752**
*Heterotrait-monotrait ratio (HTMT) criterion*			
	Original	HTMT Int_5.0%_	HTMT Int_95.0%_
INNOVATION ORIENTATION → PERFORMANCE	0.544	0.339	0.719
INNOVATION ORIENTATION → SUCCESS	0.633	0.423	0.799
SUCCESS → PERFORMANCE	0.840	0.718	0.935

With respect to collinearity problems, the variance inflation factor coefficients of all the structural relationships were calculated, and in no case was high collinearity perceived, all the VIF being notably lower than 3. Assessment of the model’s explanatory capacity with respect to the dependent variables placed the focus on the R^2^ coefficient of the endogenous constructs – success and performance. In both cases, the explanatory capacity was in the moderate range (Chin, 1998), highlighting that the model can explain more than 50% of the variance of the performance of the NGDOs. In the decomposition of R^2^, the explanatory capacity of success in new projects stood out over performance.

With respect to the path coefficients (standardized regression coefficients between the latent variables), all three have a positive sign, which confirms the sign of the relationships leading the hypotheses of this study. There is a weak relationship between innovation orientation and performance, while the relationships between innovation orientation and success, and between success and performance, are far more robust. This suggests that Hypotheses 2 and 3 might be confirmed, while Hypothesis 1 seems less consistent. The study of the statistical significance of these coefficients was carried out by a random resampling process (bootstrapping; Hair et al., 2011) of 5,000 samples. Both the t-statistic test and the analysis of the confidence intervals showed that in the case of the innovation orientation/performance relationship, the null hypothesis that proposed that the value of the path coefficient is zero cannot be rejected. This implies that the H1 of our study should be rejected since it cannot be ruled out that the relationship between innovation orientation and performance has a regression coefficient equal to zero. In the same way that H1 is rejected, we can accept H2 and H3 from verifying the statistical significance of the path coefficients of these relationships.

The result of the effect size analysis was coherent with the above. The relationship featured in H1 in this study presented a small effect size, while the effect sizes of the innovation orientation/success and success/performance relationships were large.

Finally, regarding the model’s goodness-of-fit indicators, in the present study, we have tried to assess the consistency and goodness of the model in two ways: through fit indicators and through inferential statistics (Henseler et al., 2016). The fit indicator used (SRMR) indicates a good model fit (Hu and Bentler, 1998). With respect to the inferential statistics, a fit test based on bootstrapping (Dijkstra and Henseler, 2015; Henseler et al., 2016; Henseler, 2017) was performed for the SRMR, d_ULS, and d_G parameters. In all three tests, the original value of the statistic was lower than the value of the upper limit in the confidence intervals, both 95 and 99%, which indicates that it was a good fit and the model cannot be rejected (Henseler et al., 2016).

### Analysis of the Mediation Effect

The rejection of Hypothesis 1 shows that it cannot be guaranteed that the relationship between innovation orientation and performance is significant. Discarding the presence of this direct relationship raises the possibility of assessing whether there is a mediatory effect in the relationship of innovation orientation and performance, a mediation occurring through the success of new projects. This possibility underlies Hypothesis 4 of the present study, which presupposes a mediating effect of the success of new projects on the innovation orientation/performance relationship.

**Figure 2:**
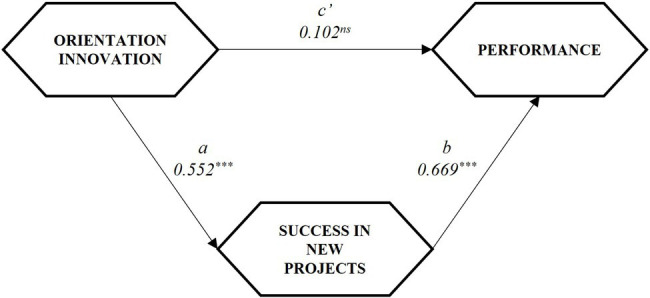
Research model: indicators for the mediation test.

The results of the mediation test are presented in Table 9 and Figure 2. The test was based on percentile bootstrapping (Hayes and Scharkow, 2013) with 5,000 samples.

**Table 9:** Indicators of the structural model analysis.

*VIF*					
INNOVATION ORIENTATION → PERFORMANCE			1.438		
INNOVATION ORIENTATION → SUCCESS			1.000		
SUCCESS → PERFORMANCE			1.438		
*Effects on the endogenous variables*					
	*Adjusted R* ^2^	*Q* ^2^	*Direct effect*	*Correlation*	*Variance explained (*%*)*
SUCCESS	0.305	0.152			**30.5**
INNOVATION ORIENTATION			0.552	0.552	30.5
PERFORMANCE	0.534	0.285			**53.4**
INNOVATION ORIENTATION			0.102	0.472	4.8
SUCCESS			0.669	0.726	48.6
*Results of the structural model*					
	*Path coeff.*	*t-value*	*Path Int*_5.0%_	*Path Int*_95.0%_	*Support*
H1: INNOV_OR → PERFORMANCE	0.102^ns^	1.169	−0.054	0.231	NO
H2: INNOV_OR → SUCCESS	0.552^***^	5.094	0.370	0.722	YES
H3: SUCCESS → PERFORMANCE	0.669^***^	9.955	0.563	0.781	YES
*Effect size (f* ^2^ *)*					
INNOVATION ORIENTATION → PERFORMANCE			0.016		
INNOVATION ORIENTATION → SUCCESS			0.438		
SUCCESS → PERFORMANCE			0.669		
*Goodness-of-fit*					
			*Int*_95.0%_		*Int*_99.0%_
SRMR	0.065		0.068		0.075
d_ULS	0.573		0.620		0.766
d_G	0.275		0.277		0.323
NFI	0.871				

***p < 0.001, based on t (9999), one-tailed test, t (0.05; 9999) = 1.645, t (0.01; 9999) = 2.327, and t (0.001; 9999) = 3.092. ns = not significant.

The results show the existence of complete mediation, insofar as the path coefficient of the direct relationship (c’) is not significant but the indirect effect (a × b) does reach significance. This total mediation is also reinforced by the high value of the VAF coefficient.

Therefore, it is assumed that H4 can be accepted, with an indirect relationship between innovation orientation and performance, a relationship mediated by the effect of success achieved in new projects or actions developed.

### Predictive Analytics

Finally, the predictive capacity of the model was analyzed (Table 9). The first perception of the predictive power of the model to foretell the behavior of the dependent variables was extracted from the Q^2^ coefficient. According to this parameter, there existed predictive capacity (Hair and Sarstedt, 2019) as all the coefficients were greater than zero, with the predictive relevance being low in the case of performance and medium for new project success.

Subsequently, the out-of-sample predictive power was measured with the PLS Predict tool (Shmueli et al. 2016). The data (Table 10) show the model’s predictive capacity for all of its items insofar as the difference between the prediction error of the model (PLS) and the prediction error of a rival model (LM) was negative in all cases.

**Table 10:** Mediation analysis.

Effects	Point estimate	Percentile		Bias-Corrected		VAF
		Int_5.0%_	Int_95.0%_	Int_5.0%_	Int_95.0%_	
H1: c’	0.102^ns^	−0.054	0.231	−0.057	0.232	
a × b	0.369^***^	0.231	0.531	0.224	0.524	78.2%

***p < 0.001, based on t (9999), one-tailed test, t (0.05; 9999) = 1.645, t (0.01; 9999) = 2.327, and t (0.001; 9999) = 3.092. ns = not significant.

**Table 11:** Analysis of the model’s predictive power.

*Construct prediction summary*									
	*PLS Model*								
	RMSE	MAE	Q^2^_predict						
PERFORMANCE	0.976	0.718	0.182						
SUCCESS	0.955	0.660	0.237						
*Dimension prediction summary*									
	*PLS Model*			*LM*			*[PLS-LM]*		
	RMSE	MAE	Q^2^_predict	RMSE	MAE	Q^2^_predict	ΔRMSE	ΔMAE	Q^2^_predict
Op. effectiveness	0.972	0.739	**0.085**	1.053	0.802	−0.073	−0.080	−0.062	0.157
Visibility	0.968	0.791	**0.093**	1.011	0.827	0.011	−0.043	−0.036	0.082
Impact	0.935	0.643	**0.157**	0.981	0.709	0.072	−0.046	−0.066	0.085
Fund-raising	0.983	0.808	**0.053**	1.033	0.856	−0.044	−0.049	−0.049	0.097
*Indicator prediction summary*									
	*PLS Model*			*LM*			*[PLS-LM]*		
	RMSE	MAE	Q^2^_predict	RMSE	MAE	Q^2^_predict	ΔRMSE	ΔMAE	Q^2^_predict
SUCCESS_4	1.453	1.170	**0.106**	1.492	1.186	0.058	−0.039	−0.015	0.048
SUCCESS_5	1.134	0.882	**0.164**	1.228	0.964	0.020	−0.094	−0.082	0.144
SUCCESS_3	1.205	0.879	**0.048**	1.254	0.931	−0.032	−0.050	−0.052	0.080
SUCCESS_1	1.016	0.716	**0.025**	1.106	0.786	−0.156	−0.090	−0.070	0.181
SUCCESS_2	1.107	0.801	**0.212**	1.174	0.865	0.114	−0.067	−0.064	0.098
*Measurement of skewness of prediction errors*									
	Skewness		*Error used*		*Δ[PLS-LM]*		*Predictive power*		
Op. effectiveness	−1.017		MAE		**−0.062**		YES		
Visibility	−0.822		RMSE		**−0.043**		YES		
Impact	−0.865		RMSE		**−0.046**		YES		
Fund-raising	−0.808		RMSE		**−0.049**		YES		
SUCCESS_4	−0.882		RMSE		**−0.039**		YES		
SUCCESS_5	−0.905		RMSE		**−0.094**		YES		
SUCCESS_3	−0.726		RMSE		**−0.050**		YES		
SUCCESS_1	−0.940		RMSE		**−0.090**		YES		
SUCCESS_2	−0.909		RMSE		**−0.067**		YES		

## Discussion and Conclusion

There is no doubt that the current panorama of international cooperation for development is determined by the framework of the Sustainable Development Goals for the period 2015–2030. This new development agenda poses an important challenge for all the actors involved in the promotion of the development and aims to apply effective responses to major global problems in the current context. Nonetheless, at the same time, it requires an important redefinition of the roles that the different actors of the international cooperation system must play, to the extent that their strategies and actions need to be adapted to the reality of international relations and the current economic scenario.

In this way, NGDOs are facing a transcendental moment of change to adapt to the role they must play in the new Development Agenda, a role still to be defined in many cases. This raises the need for a major reflection on their geographical areas of action, the principles and causes to defend, audiences to address, and intervention tools. Nonetheless, it is essential that they simultaneously preserve a good part of their essence and the distinctive aspects that have served to underpin their strengths as actors in the system.

On many occasions, this type of challenge can lead to a logical dizziness or misgiving to which NGDOs are no strangers. The objective of this study was to contribute to reducing obstacles to innovation in the entities that work toward attaining the SDGs by offering evidence of the positive impact that innovation has on the success and performance of these entities.

This research attempts to provide evidence, based on quantitative data, on the importance of a culture of innovation in a context – international development cooperation organizations – where most previous research has been based on case studies or qualitative analysis. In this way, we have sought to contribute to extending the development of predictive causal models in the field of entities directly involved in the achievement of the UN sustainable development goals. The proposed model was in general able to explain a good part of the variance of the endogenous constructs considered. In addition, regarding the hypotheses posited, three of the four were validated, all three showing robust statistical significance.

For the non-validated hypothesis specifically, it was not possible to verify that innovation orientation contributes directly to achieving greater performance (H1). In this sense, Narver et al. (2004) observed that the relationship between innovation orientation and results may lose significance with the inclusion of other variables. Nonetheless, it is not considered that, in the area of NGDOs, the predisposition to innovate is not a distinctive and interesting management characteristic. On the contrary, the verification of the existence of a mediating effect (Hypothesis 4 of the study) suggests that – consistent with Hult et al. (2004) – innovation orientation is a key factor insofar as it contributes indirectly to improving performance, through the substantial improvement it generates in the potential success of future interventions and projects that the organization designs.

This conclusion is reinforced by the consistency of Hypotheses 2 and 3. Success in new projects has a direct and positive impact on performance (H3), with success also being the model variable that contributes the most to explaining the variance of the endogenous variable performance. In fact, success explains 48.6% of the variance of performance. These findings are consistent with the suggestions provided by Datta et al. (2019) or Farooq et al. (2021). According to these authors, achieving success in new projects reduces uncertainty in an organization’s environment, thus promoting the achievement of higher organizational performance.

The data suggest that NGDOs that manage to improve the design of their interventions end up achieving a greater degree of impact, fund-raising, operational efficiency, and visibility. In the search for the antecedents of success, it was found that its improvement is positively impacted by innovative orientation (H2). In this sense, the data show that innovation orientation predicts 30.5% of the variance of success. This result confirms the key idea held by different authors (Zhang et al., 2015; Gundry et al., 2016; Stock and Schnarr, 2016), according to which, without a clear innovation orientation it is difficult to obtain success in new projects.

In view of this scenario, the present study concludes that those organizations which manage to maintain excellence and success in their future interventions will be those that can achieve a greater performance, which includes, among other factors, the degree of fulfillment of the organization’s mission. Based on the results of the model, our recommendation is that NGDO managers try to encourage within their organizations a culture of sustainable innovation over time, giving it the importance it merits as a precursor to success and an indirect precedent of the organization’s performance. A result will be to foster a climate that favors the generation of ideas, discussion of alternatives, commitment to seeking new ways of doing things, attention to the environment to learn new processes and to identify good practices, and the capacities needed for all these actions to be reflected in the design of new interventions or projects. The promotion of these processes can generate favorable impacts in different dimensions, both those that are more operational, such as the number of subsidized projects or the private financing achieved, as well as other more complex ones, such as the ability to adapt to the new realities of the Agenda, the visibility of the entity, or the degree of fulfillment of the mission that includes the reason for being of these entities. We hope that this work will encourage managers of international cooperation entities to establish routines and dynamics that will gradually generate a culture of innovation in these organizations, recognizing that these processes have a positive impact on the fulfillment of their organizational mission.

## Data Availability Statement

The raw data supporting the conclusions of this article will be made available by the authors, without undue reservation.

## Ethics Statement

Ethical review and approval were not required for the study on human participants in accordance with the local legislation and institutional requirements. Written informed consent for participation was not required for this study in accordance with the national legislation and the institutional requirements.

## Author Contributions

VV-A, CG-C, and MB-M contributed to the theoretical development of the work and construction of the model, and wrote sections of the manuscript. VV-A has contributed in data capture and data analysis. All authors contributed to manuscript revision, read, and approved the submitted version.

## Funding

This research was co-financed by the Junta de Extremadura and the European Regional Development Fund (ERDF), project code GR18027.

## Conflict of Interest

The authors declare that the research was conducted in the absence of any commercial or financial relationships that could be construed as a potential conflict of interest.

## Publisher’s Note

All claims expressed in this article are solely those of the authors and do not necessarily represent those of their affiliated organizations, or those of the publisher, the editors and the reviewers. Any product that may be evaluated in this article, or claim that may be made by its manufacturer, is not guaranteed or endorsed by the publisher.
